# Development of Porous Pt Electrocatalysts for Oxygen Reduction and Evolution Reactions

**DOI:** 10.3390/molecules25102398

**Published:** 2020-05-21

**Authors:** Marika Muto, Mayumi Nagayama, Kazunari Sasaki, Akari Hayashi

**Affiliations:** 1 Department of Hydrogen Energy Systems, Kyushu University, 744 Motooka, Nishi-ku, Fukuoka 819-0395, Japan; mmjb004@gmail.com (M.M.); sasaki@mech.kyushu-u.ac.jp (K.S.); 2 Coevolutionary Research for Sustainable Communities (COI-C2RSC), Kyushu University, 744 Motooka, Nishi-ku, Fukuoka 819-0395, Japan; nagayama.mayumi.630@m.kyushu-u.ac.jp; 3 International Research Center for Hydrogen Energy, Kyushu University, 744 Motooka, Nishi-ku, Fukuoka 819-0395, Japan; 4 NEXT-FC, Kyushu University, 744 Motooka, Nishi-ku, Fukuoka 819-0395, Japan; 5 Q-PIT, Kyushu University, 744 Motooka, Nishi-ku, Fukuoka 819-0395, Japan

**Keywords:** oxygen reduction, oxygen evolution, PEM fuel cell, PEM water electorolyzer, durability, porous structure, carbon-free

## Abstract

Porous Pt electrocatalysts have been developed as an example of carbon-free porous metal catalysts in anticipation of polymer electrolyte membrane (PEM) fuel cells and PEM water electrolyzers through the assembly of the metal precursor and surfactant. In this study, porous Pt was structurally evaluated and found to have a porous structure composed of connected Pt particles. The resulting specific electrochemical surface area (ECSA) of porous Pt was 12.4 m^2^ g^−1^, which was higher than that of commercially available Pt black. Accordingly, porous Pt showed higher oxygen reduction reaction (ORR) and oxygen evolution reaction (OER) activity than Pt black. When the activity was compared to that of a common carbon-supported electrocatalyst, Pt/ketjen black (KB), porous Pt showed a comparable ORR current density (2.5 mA cm^−2^ at 0.9 V for Pt/KB and 2.1 mA cm^−2^ at 0.9 V for porous Pt), and OER current density (6.8 mA cm^−2^ at 1.8 V for Pt/KB and 7.0 mA cm^−1^ at 1.8 V), even though the ECSA of porous Pt was only one-sixth that of Pt/KB. Moreover, it exhibited a higher durability against 1.8 V. In addition, when catalyst layers were spray-printed on the Nafion^®^ membrane, porous Pt displayed more uniform layers in comparison to Pt black, showing an advantage in its usage as a thin layer.

## 1. Introduction

Electrocatalysts composed of noble metal nanoparticles dispersed on carbon supports are commonly used in polymer electrolyte membrane (PEM) fuel cells and PEM water electrolyzers. Such dispersion of metal nanoparticles leads to high catalytic activity, even with small amounts of noble metals. However, carbon supports are sometimes not usable. For example, in the case of PEM water electrolyzers, carbon supports are highly corroded under high potential anodic conditions (over 1.8 V), which are required to obtain a practical current density of 1 A cm^−2^ [1,2]. Even in the PEM fuel cell system, carbon oxidation at the cathode is serious at a locally elevated potential (over 1.5 V) during the start/stop cycling of fuel cell vehicles [3,4]. Additionally, even at the anode catalyst, carbon corrosion has been reported in the situation of fuel starvation, where cell reversal occurs and the anode potential is over 1.5 V [5]. Not only are the carbon supports themselves damaged, but the metal nanoparticles also lose their support, leading to the agglomeration of particles and loss of their catalytic activity [6].

Even though problems related to the durability of carbon supports have been reported, increasing the electroactive surface area without a carbon support is rather challenging. A large quantity of novel metal catalysts with a low surface area should generally be employed without carbon supports, which increases their cost [7]. For that reason, the improvement of electrocatalysts without carbon supports has been extensively studied. In some cases, metal oxide supports have been used, but this still results in a low electronic conductivity in comparison to carbon, even though durability is high [8,9,10,11,12,13,14]. For example, although the oxygen reduction reaction (ORR) mass activity of Pt/doped tin oxide at 0.85 V has been reported to be around 600 A g^−1^, which is comparable to that of Pt-deposited carbon, under half-cell measurements in solution the ORR activity at 0.9 V, which is common for standard comparisons, is expected to be low [10]. In other cases, catalysts made of only metal nanoparticles have been studied owing to their high surface area [15,16,17,18]. Although nanoparticles show high catalytic activity as powder, they cannot usually maintain their structure in actual devices and mostly result in low performance. However, when these nanoparticles are connected to each other, they can maintain their porous structure, even in actual devices, and a current–voltage performance comparable to that of PEM fuel cells exhibited by Pt supported on carbon at 0.8 V, with a value of 200 mA cm^−1^, has been reported [17]. Among many carbon support-free catalysts, one of the most successful catalysts is 3M’s nanostructured thin film (NSTF) catalyst, which is a pure, organic molecular solid in the form of a crystalline whisker coated by metal catalysts. The NSTF catalyst has shown 2–3 times higher mass activity at 0.9 V, with a value of over 600 A g^−1^, and a higher durability than Pt supported on a carbon support [19].

We are rather interested in developing metal-only catalysts and improving their structure as devices. In our prior studies, mesoporous carbon supports were developed through a simple one-pot reaction that involved heating a mixture of a surfactant and carbon precursors for PEM fuel cells, and high performance and durability were achieved in accordance with their mesoporous structure [20,21,22,23,24,25]. The porous structure was also sustained, even after being built into a device. We are now trying to apply the concept of mesoporous structures to metal-only electrocatalysts with the aim of increasing the surface area and reducing the mass transfer loss of water and gases in the PEM fuel cell and water electrolysis system based on the porous structure. In addition, conductivities superior to those of metal oxide supports are expected for all metallic compositions.

In this paper, porous Pt has been synthesized as one example of a porous metal catalyst by employing a Pt precursor and a surfactant. Step-by-step heat treatments were applied to reduce the Pt precursor and remove the surfactant. Resulting carbon-free porous Pt was structurally evaluated, and its electrochemical activity toward the ORR and oxygen evaluation reaction (OER) was studied in detail. Additionally, a catalyst layer of porous Pt was prepared using a spray printing method and its structure was evaluated.

## 2. Results and Discussion

### 2.1. Heat Treatment and Characterization of Porous Pt

The heat treatment conditions for porous Pt were controlled in order to remove the residual derived from the surfactant and metal precursor. In the case where the sample was calcined at 210 °C for 3 h and 240 °C for 3 h for the thermal reduction of Pt(acac)_2_ and also heat-treated at 300 °C for 1 h for decomposition of the surfactant under a nitrogen atmosphere, a sticky product was obtained owing to the residual surfactant. Therefore, the heat treatment condition for decomposing the surfactant was altered to 400 °C for 3 h. As a result, a dry powder was obtained. However, based on thermogravimetric (TG) analysis, a weight loss of 62.3% was further observed at around 200 °C (see Supplementary Materials Figure S1a), indicating that the residual surfactant remained in this condition. In order to further remove the residual surfactant completely, additional heat treatment at 200 °C under humidified nitrogen was conducted. Consequently, the weight loss was minimized to 3.6% after calcining for 10 min with this condition (see Figure S1b), resulting in successful removal of the surfactant.

**Figure 1:**
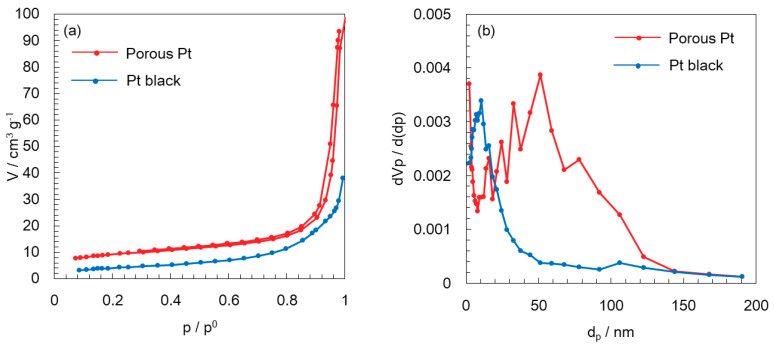
(**a**) Nitrogen adsorption/desorption isotherms and (**b**) corresponding pore distributions of porous Pt and Pt black.

Porous Pt was characterized by nitrogen sorption in comparison to commercial Pt black. Figure 1a,b show nitrogen adsorption/desorption isotherms and corresponding pore size distributions, respectively. The Brunaure–Emmett–Teller (BET) specific surface area of porous Pt was 32.4 m^2^ g^−1^, whereas that of commercially available Pt black was 14.3 m^2^ g^−1^, which was slightly low compared to the value of 25 m^2^ g^−1^ or less given by Sigma-Aldrich (St. Louis, MO, USA). Porous Pt showed mesopores consisting of less than 100 nm in diameter and micropores, while Pt black mostly exhibited small mesopores of less than 30 nm, with relatively fewer micropores. Since Pt black consists of nanoparticles not connected to each other and does not contain pores within the particles, the size of 30 nm reveals the particle size and interparticle pore size achieved by these nanoparticles.

**Figure 2:**
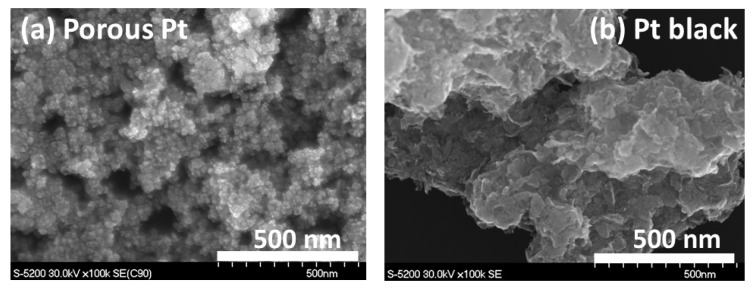
SEM images of (**a**) porous Pt and (**b**) Pt black.

In order to view the nanostructure of porous Pt and Pt black, SEM images were observed and are shown in Figure 2a,b, respectively. As seen in Figure 2a, pores consisting of connected Pt particles were confirmed. Even though porous Pt does not have a common ordered porous structure like that of zeolite, it shows pores, displaying a “porous structure” required for device application [17]. The domain size of this porous structure was estimated to range from 500 nm to 2 μm based on SEM images with a low magnification. As seen in SEM images with a higher magnification (Figure S2), the size of the average particles in porous Pt was ca. 20 nm, while Pt black showed much smaller primary particles, which were less than 10 nm. However, in the case of Pt black, much denser aggregates of such Pt particles were observed, as shown in Figure 2b.

Pt 4f XPS spectra were recorded to evaluate the surface chemical states. Both porous Pt and Pt black had two peaks at 71.1–71.3 and 74.4–74.6 eV of binding energy, corresponding to Pt4f_7/2_ and Pt4f_5/2_, respectively. Those two peaks indicated that both materials mostly contained the metallic Pt (0) surface [26]. Although oxygen bonding to the Pt surface was also indicated in the XPS spectra, this was mostly likely due to oxygen adsorption that occurred when transferring the sample in the air. This kind of oxygen cannot be easily removed unless in-situ XPS methods, such as EC-XPS [27], are applied.

XRD patterns were also analyzed (Figure S3). Both porous Pt and Pt black showed typical metallic platinum peaks [28], consisting of Pt (111) at 39.8°, Pt (200) at 46.2°, and Pt (220) at 67.4°, which matched the XPS result.

### 2.2. Electrochemical Analyses of Oxygen Reduction and Evolution Reactions

**Figure 3:**
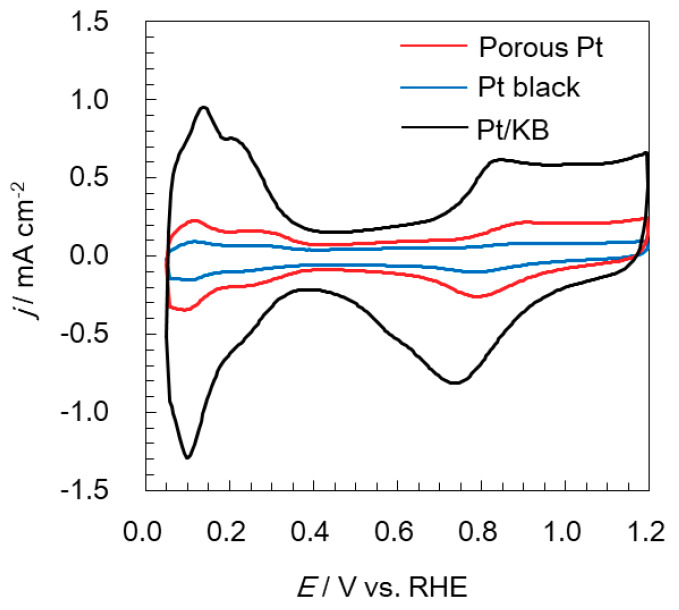
Cyclic voltammograms of porous Pt, Pt black, and Pt/ketjen black (KB).

Cyclic voltammograms of porous Pt and Pt black were recorded and are shown in Figure 3. Pt/ketjent black (KB) was also analyzed and compared as a standard electrocatalyst. The electrochemical surface area (ECSA) of porous Pt was 12.4 m^2^ g^−1^. The ECSA of Pt/KB was 76.5 m^2^ g^−1^, which stayed at the low end of reported values (70–100 m^2^ g^−1^) [29,30,31], suggesting the formation of a non-uniform thin film. Even though the film formation should be further optimized, with the films made under the same condition, Pt/KB has an ECSA that is six times higher than that of porous Pt. This is reasonable because Pt/KB has highly dispersed 2-nm Pt nanoparticles on the carbon support [32], but porous Pt consists of aggregates of much larger Pt particles. Pt black showed a lower ECSA (4.7 m^2^ g^−1^) than porous Pt. As shown in Figure 2b and Figure S2b, Pt black is composed of dense aggregates, even though primary particles are as small as several nanometers. The structural difference most likely leads to different ECSA. Additionally, cyclic voltammograms of three catalysts, where the current is normalized to the specific surface area of Pt, are shown in Figure S4. The current of Pt/KB becomes smaller than that of porous Pt and Pt black, suggesting that porous Pt and Pt black have higher activity than Pt on KB.

**Figure 4:**
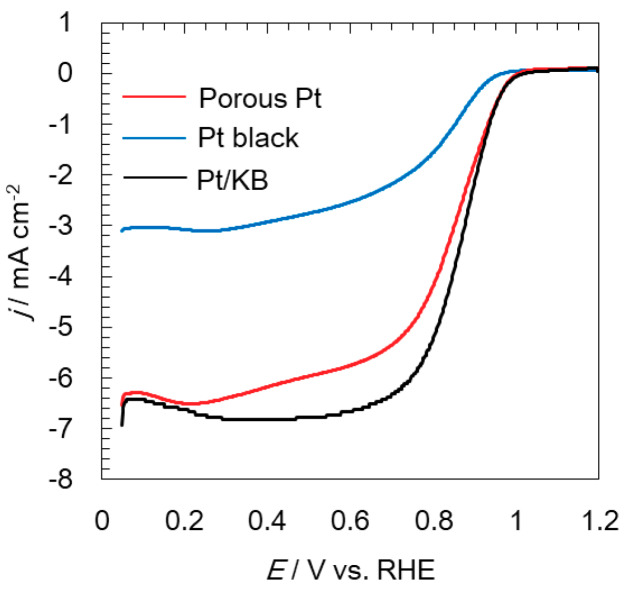
Linear sweep voltammograms showing the oxygen reduction reaction (ORR) of porous Pt, Pt black, and Pt/KB at 1600 rpm under oxygen saturation.

The ORR activities of all electrocatalysts were evaluated at a rotating speed of 1600 rpm and are shown in Figure 4. Pt had equivalent ORR activity to Pt/KB, even though a slightly slow ORR current increase was seen for porous Pt. The values of the current density at 0.9 V were 2.5 and 2.1 mA cm^−2^ for Pt/KB and porous Pt, respectively. The reported ORR activity for Pt/C is even higher, for example 3 mA cm^−2^ [30]. ORR mass activity is commonly calculated through the kinetic current, which is the current at infinite speed extrapolated from a Koutecky–Levich plot, by measuring the ORR polarization curves for different rotation speeds. In this experiment, only one rotational speed was tried. Therefore, another method of calculating the kinetic current using the limiting current at 0.4 V [33] was used. The values of the mass activity at 0.9 V were 230 and 190 A g^−1^ for Pt/KB and porous Pt, respectively. The reported ORR mass activity for Pt/C has the range but is mostly between 200 and 500 A g^−1^ [29,30]. The lower values of ORR current and ORR mass activity in this work is most likely due to the non-uniform thin film acting as a working electrode; the development of uniform thin films is required to precisely discuss ORR activity.

**Figure 5:**
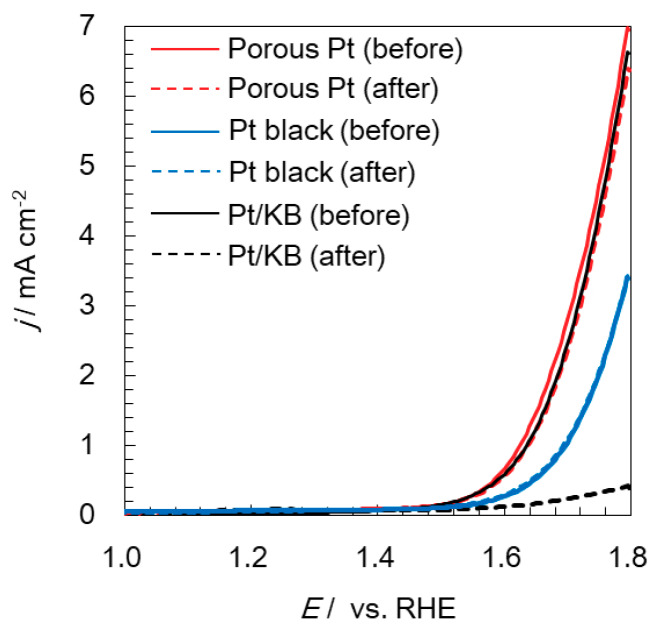
Linear sweep voltammograms showing oxygen evolution reaction (OER) of porous Pt, Pt black, and Pt/KB at 1600 rpm under nitrogen saturation. Solid and dotted lines show before and after the durability test, respectively.

The OER activities of three electrocatalysts were evaluated at a rotating speed of 1600 rpm and are shown in Figure 5 with solid lines. Porous Pt had an initial OER current equivalent to that of Pt/KB (6.8 to 7.0 mA cm^−2^ at 1.8 V), while Pt black revealed a lower OER current (3.4 mA cm^−2^ at 1.8 V) than the other two catalysts. When the reported OER activity of Pt catalysts is considered, Pt bulk and nanoparticles show an OER current density of about 4 and 10 mA cm^−2^ at 1.8 V, respectively [34], which reasonably match our results. We believe that the OER activity in the solution half-cell set-up remains constant if enough active surface area is available.

### 2.3. Electrochemical Durability of Catalysts at a High Potential

In order to evaluate durability against a high potential, the anode potential at PEM water electrolyzers was considered, since it is a more severe condition than the cathode potential at PEM fuel cells. Although the protocol for the durability examination of PEM fuel cells has been well established [4], the protocol for PEM water electrolyzers has not been developed. Therefore, the potential of 1.8 V, which is the voltage required to obtain the practical current density of 1 A cm^−2^, was applied to evaluate the durability in this study. Among the three samples, only Pt/KB was found to continuously lose OER current. Voltammograms of the three samples after the durability test were developed and are indicated with dotted lines in Figure 5. As can be seen in Figure 5, Pt/KB lost most of its OER activity by just applying 1.8 V for 10 min, owing to carbon corrosion that occurred at 1.8 V, while both porous Pt and Pt black maintained the initial OER activity according to the carbon-free condition. A similarly high durability can also be found in the literature for support-free catalysts, even though the condition of durability examination is slightly different [2,17,35]. For PEM fuel cells, current–voltage curves with the cathode of connected metal particles at 80 °C did not change much after 10,000 cycles of the start–stop durability test [17]. For PEM water electrolyzers, the changes in the voltage at 2 A cm^−2^ under 60 °C [2] or 500 mA cm^−2^ under 80 °C [35] were monitored, and the increase was less than 0.02 V during the 300 h test. Therefore, even though a longer test is required to evaluate the durability of porous Pt, an advantage of a carbon-free catalyst was experimentally observed in this study. Regarding the decrease in the current density observed in Figure 5, it was found to be a reversible change. Such reversible loss probably comes from the fact that the unsuccessful detachment of generated oxygen bubbles decreases the mass transfer capability of water on the electrode surface [36].

### 2.4. Evaluation of Catalyst Layers

**Figure 6:**
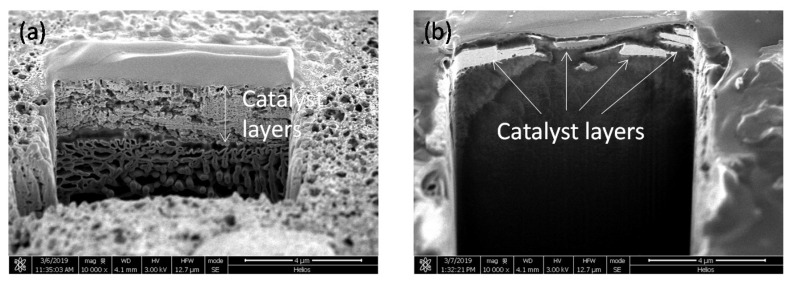
Cross section SEM images of (**a**) porous Pt and (**b**) Pt black layers.

Catalyst layers of porous Pt and Pt black were spray-printed on the Nafion^®^ membrane. Pt-loading was fixed to 0.50 mgPt/cm^2^. The cross section of catalyst layers was observed using Focused Ion Beam (FIB)-SEM and is shown in Figure 6. Although a uniform thin layer of porous Pt with an average thickness of 2.4 μm was observed, as shown in Figure 6a, in the case of Pt black, no continuous layer was seen, as shown in Figure 6b. Even though relatively high catalyst loading is observed for water electrolysis [37,38], the future direction for PEM fuel cells lies in reducing the catalyst amount, for example to 0.10 mgPt/cm^2^ or less. The formation of thin films will be more difficult when large-scale manufacturing methods of thin layers are considered. A high void volume is also important for enhancing the electrocatalytic activity [39]. Consequently, porous Pt has an advantage in its usage as a thin film, and the performance and durability of the new porous Pt catalyst-based membrane/electrode assembly will be characterized in a future study.

## 3. Materials and Methods

### 3.1. Materials

Platinum (II) acetylacetonate (Pt(acac)_2_), 5 N hydrochloric acid (HCl), ethanol, 2-propanol, and 0.1 M perchloric acid (HClO_4_) were all purchased from Wako Pure Chemical Industries Ltd. (Osaka, Japan) Pluronic^®^ F127 was obtained from Sigma-Aldrich. 5% Nafion^®^ dispersion solution and Nafion^®^ 117 were obtained from Dupont (Wilmington, DE, USA). Milli-Q water was used in all cases. Those chemicals were used without any further purification.

For the electrochemical evaluation, Pt black and Pt/KB (TEC10E50E) were used as standard electrocatalysts for the comparison in this study and obtained from Wako Pure Chemical Industries, Ltd. and Tanaka Kikinzoku Kogyo K.K. (Tokyo, Japan), respectively.

### 3.2. Synthesis of Porous Pt

Pluronic^®^ F127 and Pt(acac)_2_ were used as a template to form a porous structure and as a metal precursor, respectively. Typically, 0.4275 g of Pluronic^®^ F127 was dissolved in a mixture of water/ethanol/HCl (2.175 g/2.875 g/75 μL). Then, 0.675 g of Pt(acac)_2_ was added to this solution. This mixture was stirred at 30 °C for 6 h, kept at room temperature for 6 h, and dried at 80 °C for 6 h in the oven. After further heat treatment of the resulting powder, porous Pt materials were obtained. The heat treatment conditions, such as the temperature and atmosphere, were studied to optimize the porous structure and are fully discussed in the “Results and discussion” section.

### 3.3. Material Characterization

For material characterization, nitrogen sorption measurements were carried out using BELSORP-mini II-VS (MicrotracBEL Corp. Osaka, Japan). Before the measurement, samples were pre-treated at 200 °C under vacuum for 2 h. The specific surface area was calculated by a BET method using the adsorption area. The Barrett Joyner Hallenda (BJH) method was also applied to estimate the pore size distribution.

Thermogravimetric (TG) analyses were conducted using Thermo Plus Evo2 (Rigaku, Tokyo, Japan). Measurements were carried out in the air from room temperature to 400 °C via raising the temperature by 4 °C/min.

SEM observations were performed using S-5200 (Hitachi High-Tech, Tokyo, Japan), with the accelerating voltage of 30 kV. Studies with X-ray photoelectron spectroscopy (XPS) and X-ray diffraction (XRD) were also conducted using Kratos Axis Ultra (Shimadzu, Kyoto, Japan) and RINT-UltimaIII/PSA (Rigaku), respectively.

### 3.4. Electrochemical Analyses

The dispersion containing electrocatalysts (4.3 mg), water (0.35 mL), and 2-propanol (2.56 mL) was mixed using an ultrasonic homogenizer and drop-cast onto a glassy carbon (GC) rod (the diameter: 5 mm, Tokai Fine Carbon Co., Ltd. (Tokyo, Japan)), and dried at room temperature for use as a working electrode. The Pt loading was kept to 17.3 µg cm^−2^ in all cases. In this study, a Nafion^®^ dispersion was not used as a binder in order to simply observe electrochemical characteristics of catalysts without a Nafion^®^ effect. The fact that a binder is not essential for adhesion of the catalyst to the GC and that low-loaded ionomer free catalyst (≤~18 μgPt cm^−2^) adheres with a sufficient strength during measurements for ORR activity evaluation at room temperature has previously been reported [29].

Electrochemical measurements were performed using a common half-cell set-up in the solution. A potentiostat (HZ-7000, Hokuto Denko, Tokyo, Japan) was used for all electrochemical measurements. Besides the working electrodes, Ag|AgCl in a saturated KCl aqueous solution and Pt wire were utilized as reference and counter electrodes, respectively. Measurements were taken in 0.1 M HClO_4_ under nitrogen atmosphere with the scan rate of 50 mV s^−1^. ORR and OER activities were evaluated at 1600 rpm under the saturation of oxygen and nitrogen, respectively. In this study, all potentials were converted to a reversible hydrogen electrode (RHE), and IR correction was applied for OER evaluation by actually measuring the solution resistance using a potentiostat. Furthermore, in order to observe the durability against the anodic condition of the water electrolysis, the potential of 1.8 V vs. RHE was applied for 10 min, and OER activities before and after applying such potential were evaluated.

### 3.5. Evaluation of Catalyst Layers

Catalyst layers were prepared by employing a common spray printing method. The slurry consisting of porous Pt (or Pt black) (81.5 mg), ethanol (1764 µL), 5% Nafion^®^ dispersion solution (919 µL), and MilliQ water (196 µL) was thoroughly stirred by an ultrasonic homogenizer. In this condition, the ratio of ionomer and catalyst was fixed to 0.33. This slurry was spray-printed on Nafion^®^ 117 by a spray printing system (Nordson, Westlake, OH, USA). Catalyst layers were made into a 1 cm × 1 cm square. Pt-loading was fixed to 0.50 mgPt cm^−2^. The cross section of catalyst layers was observed using FIB-SEM (Helios 600, FEI Company, Hillsboro, OR, USA). FIB processing was carried out at an accelerating voltage of 30 kV and a beam current of 0.4 nA.

## 4. Conclusions

A carbon-free porous Pt electrocatalyst was successfully prepared using the assembly of the metal precursor and surfactant under the optimized heat treatment condition. The structure of porous Pt was found to be composed of connected Pt particles with a size of 20 nm, leading to a relatively high BET surface area of 32.4 m^2^ g^−1^, in comparison to a commercial Pt black electrocatalyst, which has a value of 14.3 m^2^ g^−1^. The ECSA of porous Pt was 12.4 m^2^ g^−1^, whereas that of Pt black was 4.7 m^2^ g^−1^. Accordingly, porous Pt showed higher ORR and OER activity than that of Pt black.

In comparison to a standard electrocatalyst, Pt/KB, an equivalent ORR current density (2.5 mA cm^−1^ at 0.9 V for Pt/KB and 190 mA cm^−1^ at 0.9 V for porous Pt), ORR mass activity (230 A g^−1^ at 0.9 V for Pt/KB and 2.1 A g^−1^ at 0.9 V for porous Pt), and OER current density (6.8 mA cm^−1^ at 1.8 V for Pt/KB and 7.0 mA cm^−1^ at 1.8 V) were obtained, even though the ECSA of porous Pt was only one-sixth that of Pt/KB. However, Pt/KB lost most of its OER activity during the durability test against the anode potential during water electrolysis owing to carbon corrosion, whereas porous Pt maintained its OER activity due to the effect of the carbon-free condition.

In addition, when catalyst layers were spray-printed on the Nafion^®^ membrane, porous Pt showed more uniform layers in comparison to carbon-free Pt black, demonstrating the advantage of its usage as a thin film.

Consequently, porous Pt with high ORR and OER activities and a high durability against a high potential was successfully prepared in this study and showed potential to be used as an electrocatalyst for PEM fuel cells and PEM water electrolyzers.

## Supplementary Materials

The following are available online. Figure S1: Thermogravimetric (TG) analyses of porous Pt (a) with heat treatment at 400 °C for 3 h under dry nitrogen and (b) with additional heat treatment at 200 °C for 10 min under humidified nitrogen, Figure S2: SEM images of (a) porous Pt and (b) Pt black with a higher magnification, Figure S3: XRD patterns of porous Pt (top, red) and Pt black (bottom, blue), Figure S4: Cyclic voltammograms of porous Pt (red), Pt black (blue), and Pt/KB (black), where the current is normalized to specific surface area of Pt.
