# Supplementary material for: Development of Porous Pt Electrocatalysts for Oxygen Reduction and Evolution Reactions
Source: Molecules. 2020 May 21;25(10):2398. doi: 10.3390/molecules25102398 (PMC7287804; doi:10.3390/molecules25102398)
Supplement: Supplementary file 1 [file molecules-25-02398-s001.pdf]

Development of Porous Pt Electrocatalysts for Oxygen Reduction and Evolution Reactions

Marika Muto [1], Mayumi Nagayama [2], Kazunari Sasaki [1,2,3,4] and Akari Hayashi [1,2,3,4,5,*]

[1] Department of Hydrogen Energy Systems, Kyushu University, 744 Motooka, Nishi-ku, Fukuoka 819-0395, Japan; mmjb004@gmail.com (M.M.); sasaki@mech.kyushu-u.ac.jp (K.S.)

[2] Coevolutionary Research for Sustainable Communities (COI-C²RSC), Kyushu University, 744 Motooka,
Nishi-ku, Fukuoka 819-0395, Japan; nagayama.mayumi.630@m.kyushu-u.ac.jp

[3] International Research Center for Hydrogen Energy, Kyushu University, 744 Motooka, Nishi-ku, Fukuoka 819-0395, Japan

[4] NEXT-FC, Kyushu University, 744 Motooka, Nishi-ku, Fukuoka 819-0395, Japan

[5] Q-PIT, Kyushu University, 744 Motooka, Nishi-ku, Fukuoka 819-0395, Japan

* Correspondence: hayashi.akari.500@m.kyushu-u.ac.jp

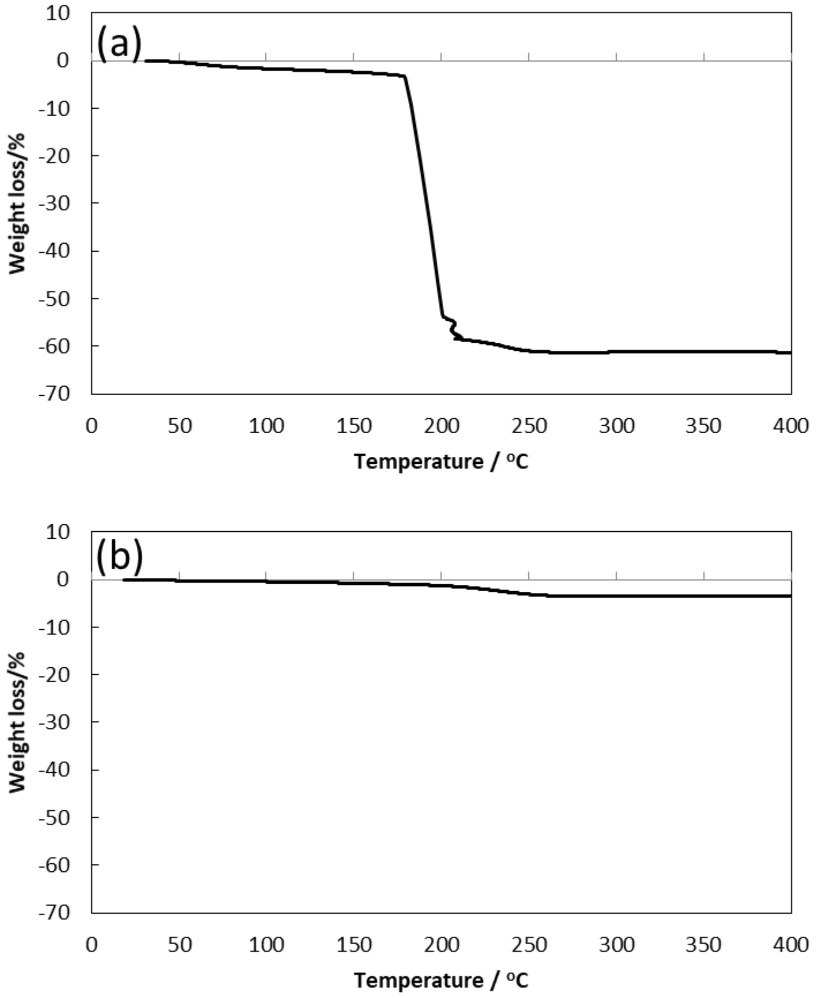

Figure S1. Thermogravimetric (TG) analyses of porous Pt (**a**) with heat treatment at 400 °C for 3 hours under dry nitrogen and (**b**) with additional heat treatment at 200 °C for 10 minutes under humidified nitrogen.

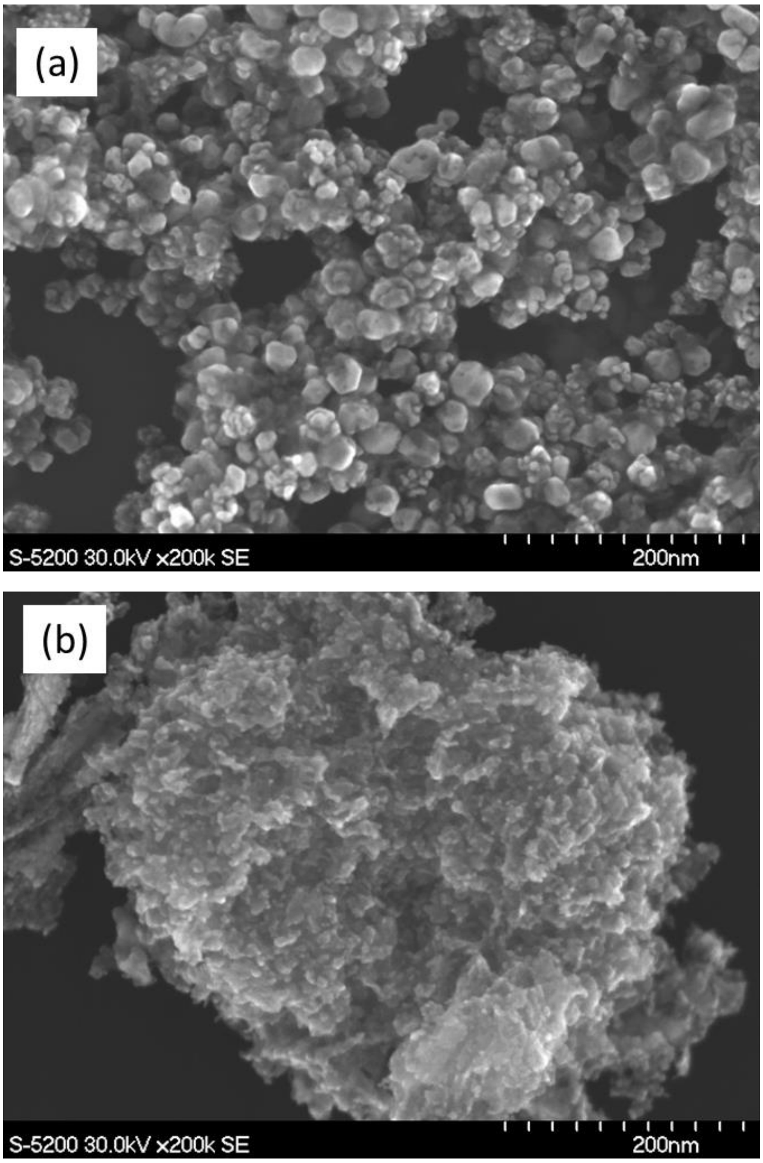

Figure S2. SEM images of (**a**) porous Pt and (**b**) Pt black with a higher magnification.

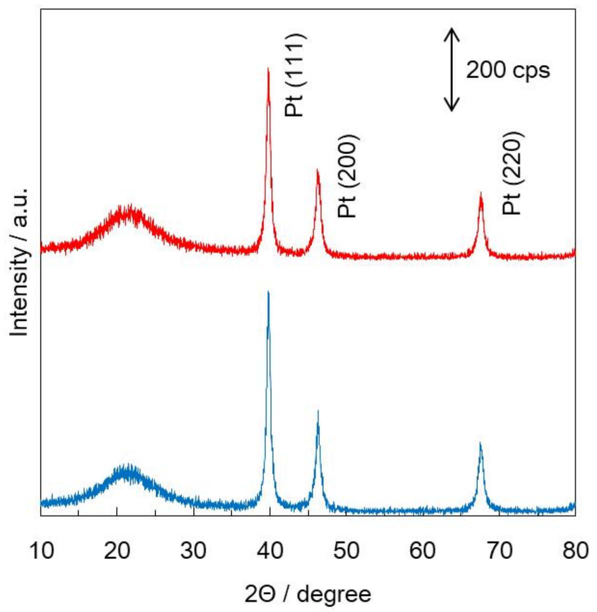

Figure S3. XRD patterns of porous Pt (top, red) and Pt black (bottom, blue).

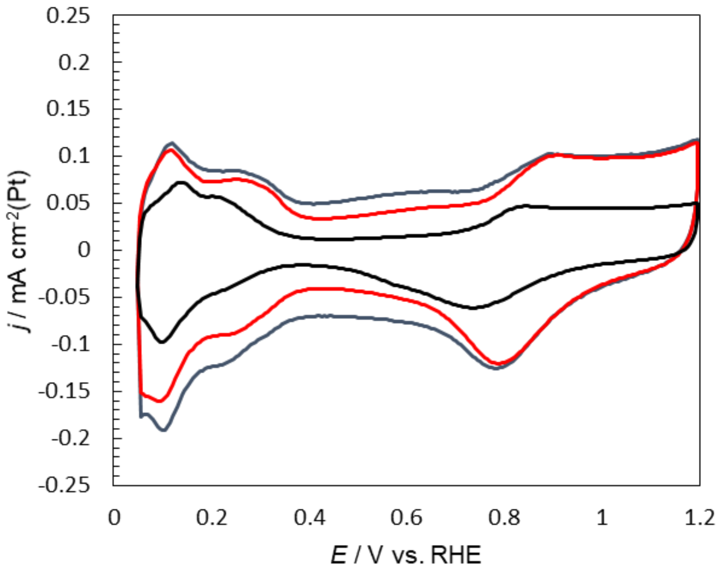

Figure S4. Cyclic voltammograms of porous Pt (red), Pt black (blue), and Pt/KB (black), where the current is normalized to specific surface area of Pt.
